# Associations of comorbid depression with cardiovascular-renal events and all-cause mortality accounting for patient reported outcomes in individuals with type 2 diabetes: a 6-year prospective analysis of the Hong Kong Diabetes Register

**DOI:** 10.3389/fendo.2024.1284799

**Published:** 2024-03-22

**Authors:** Yiu-Lam Yeung, Ka-Long Lee, Eric SH. Lau, Tsun-Fung Yung, Aimin Yang, Hongjiang Wu, Kelly TC. Wong, Alice PS. Kong, Elaine YK. Chow, Ronald CW. Ma, Theresa Yeung, Kit-man Loo, Risa Ozaki, Andrea OY. Luk, Juliana NM. Lui, Juliana CN. Chan

**Affiliations:** ^1^ Department of Medicine and Therapeutics, The Chinese University of Hong Kong, Prince of Wales Hospital, Shatin, China; ^2^ Hong Kong Institute of Diabetes and Obesity, The Chinese University of Hong Kong, Prince of Wales Hospital, Shatin, China; ^3^ Li Ka Shing Institute of Health Sciences, The Chinese University of Hong Kong, Prince of Wales Hospital, Shatin, China

**Keywords:** depression, cardiovascular-renal events, mortality, patient reported outcomes, health related quality of life

## Abstract

**Background:**

Psychosocial status and patient reported outcomes (PRO) [depression and health-related quality-of-life (HRQoL)] are major health determinants. We investigated the association between depression and clinical outcomes in Chinese patients with type 2 diabetes (T2D), adjusted for PRO.

**Methods:**

Using prospective data from Hong Kong Diabetes Register (2013-2019), we estimated the hazard-ratio (HR, 95%CI) of depression (validated Patient Health Questionnaire 9 (PHQ-9) score≥7) with incident cardiovascular disease (CVD), ischemic heart disease (IHD), chronic kidney disease (CKD: eGFR<60 ml/min/1.73m^2^) and all-cause mortality in 4525 Chinese patients with T2D adjusted for patient characteristics, renal function, medications, self-care and HRQoL domains (mobility, self-care, usual activities, pain/discomfort, anxiety/depression measured by EQ-5D-3L) in linear-regression models.

**Results:**

In this cohort without prior events [mean ± SD age:55.7 ± 10.6, 43.7% women, median (IQR) disease duration of 7.0 (2.0-13.0) years, HbA1c, 7.2% (6.6%-8.20%), 26.4% insulin-treated], 537(11.9%) patients had depressive symptoms and 1923 (42.5%) patients had some problems with HRQoL at baseline. After 5.6(IQR: 4.4-6.2) years, 141 patients (3.1%) died, 533(11.8%) developed CKD and 164(3.6%) developed CVD. In a fully-adjusted model (model 4) including self-care and HRQoL, the aHR of depression was 1.99 (95% confidence interval CI):1.25-3.18) for CVD, 2.29 (1.25-4.21) for IHD. Depression was associated with all-cause mortality in models 1-3 adjusted for demographics, clinical characteristics and self-care, but was attenuated after adjusting for HRQoL (model 4- 1.54; 95%CI: 0.91-2.60), though HR still indicated same direction with important magnitude. Patients who reported having regular exercise (3-4 times per week) had reduced aHR of CKD [0.61 (0.41–0.89)]. Item 4 of PHQ-9 (feeling tired, little energy) was independently associated with all-cause mortality with aHR of 1.66 (1.30-2.12).

**Conclusion:**

Depression exhibits significant association with CVD, IHD, and all-cause mortality in patients with diabetes, adjusting for their HRQoL and health behaviors. Despite the association between depression and all-cause mortality attenuated after adjusting for HRQoL, the effect size remains substantial. The feeling of tiredness or having little energy, as assessed by item Q4 of the PHQ-9 questionnaire, was found to be significantly associated with an increased risk of all-cause mortality after covariate adjustments. Our findings emphasize the importance of incorporating psychiatric evaluations into holistic diabetes management.

## 1 Introduction

Type 2 diabetes (T2D) is a chronic disease requiring self-care and discipline to prevent complications and premature death (1, 2). Rapid socio-economical changes in China were paralleled by a rise in T2D prevalence from 1% in 1980 to 10% in 2021 (2). Depression and diabetes (3) frequently coexist, with most of the data coming from Europeans. In the last 30 years, mental illness, especially depression, has become prevalent across Asia (4). In 2013, amongst 0.5 million Chinese participating in the China Kadoorie Biobank Project, those with major depression had 1.75 times (95%CI: 1.47–2.08) increased risk of prevalent T2D (5). In a Hong Kong clinic-based register, we reported that 18% of patients with T2D had depression (Patient health questionnaire 9 (PHQ-9) score of ≥7), which was associated with poor glycaemic control and hypoglycaemia (6), in part due to poor treatment adherence (7).

From a biological perspective, neurohormonal dysregulation associated with depression may worsen cardiovascular risk factors (8). There are multiple clinical studies that reported patients with T2D and co-morbid depression have elevated risk in experiencing CVD morbidity and mortality (9–11). In our previous study, we reported that using a diagnosis of depression registered by psychiatrist, Hong Kong Chinese patients with T2D who received specialist care for depression had more than 2 times increased risk of premature mortality and cardiovascular disease (CVD) than those without depression (12). Against a backdrop of growing burden of diabetes and depression, the Lancet Commission Report on Diabetes (1) and American Diabetes Association/European Association for Study of Diabetes (ADA/EASD) practice guidelines (13) highlighted the importance of evaluating psychosocial needs and patient-reported outcomes (PRO) including depressive symptoms, health-related quality of life (HRQoL), self-care and their inter-relationships in influencing clinical outcomes to inform practice and policies. There is paucity in evidence that establish association between depression, cardiovascular-renal outcomes and mortality that consider the influence of PROs such as HRQoL and health behaviours of patients with diabetes.

In this study, utilizing the Hong Kong Diabetes Register (HKDR) with detailed documentation of clinical profiles including PRO during structured assessment, we examined prospectively the association of depression with all-cause mortality, CVD and CKD and their associations with PRO including self-care and HRQoL in patients with T2D.

## 2 Materials and methods

### 2.1 Patients

The HKDR was established in 1995 at the Diabetes and Endocrine Centre, Prince of Wales Hospital (PWH), as a research-driven quality improvement program using structured clinical assessment (14). Using a unique identifier, HKDR was linked to a territory-wide electronic medical record system with hospitalization data and death registry for epidemiological analysis. In the present analysis, we included patients diagnosed with T2D aged ≥18 years and excluded patients with Type 1 diabetes (T1D) in the HKDR. The latter was defined by acute presentation with ketosis or requirement of continuous insulin treatment within 1 year of diagnosis, adapted from a definition of T1D in Caucasians (15). Hospitalization data was captured using international classification of disease codes (ICD-9) and causes of death by ICD-10 (14) (**Supplementary Table 1**). In 2007, we included EuroQol-5 Dimension 3 Levels (EQ5D-3L) to measure HRQoL (16) and in 2013, we included Chinese-validated PHQ-9 to measure depression (6). By 2019, 6818 patients had completed both PHQ-9 and EQ5D-3L. Amongst them, we excluded 2293 patients with (1) history of CVD [stroke, peripheral vascular disease (PVD), ischaemic heart disease (IHD)] (n=1436) and/or (2) CKD defined as estimated glomerular filtration rate (eGFR) less than 60 ml/min/1.73m^2^ (n=1181) and/or (3) incomplete responses to the PHQ-9 questionnaire (n=112). We analysed clinical outcomes in the remaining 4525 patients with T2D, of whom 4429 (97.9%) had completed all items of EQ5D-3L.

### 2.2 Baseline clinical assessment

All participants of the HKDR underwent protocol-driven assessment by trained nurses (history taking, physical examination including eye and feet and laboratory investigations including blood and urine tests) directed by case report forms. The data included sociodemographic factors, years of education, occupation, medical history, current drug use and self-care [adherence to a balanced diet, regular exercise, self-monitoring of blood glucose (SMBG), medication adherence] were documented. Physical examination included measurements of blood pressure, body weight, height, waist and hip circumference (14). After an overnight fast, blood was drawn for measurement of glycated haemoglobin (HbA1c), plasma glucose, lipid profile (total cholesterol, triglyceride, high-density cholesterol (HDL-C) and calculated low-density lipoprotein cholesterol (LDL-C) and random spot urine sample was used to measure urinary albumin-to-creatinine ratio (ACR). The Chronic Kidney Disease Epidemiology Collaboration (CKD-EPI) equation was used to derive estimated glomerular filtration rate (eGFR) (17).

### 2.3 Psychological assessment

The PHQ-9 was derived from the Diagnostic and Statistical Manual of Mental Disorders (DSM-IV) diagnostic criteria for major depression. Based on a 2-week recall period, the questionnaire consists of 9 items with a score range of 0 (not at all) to 3 (nearly every day) for each item with a total score range of 0-27. In Hong Kong Chinese patients with T2D, using semi-structured interview as reference test, our group validated a cut-off score of 7 to detect depression with optimal sensitivity and specificity (6) versus a cut-off value of 10 in most European studies (6).

PHQ-9 items Q1 (little interest or pleasure in doing things) and Q2 (feeling down, depressed or hopeless) had been validated as a short screening tool (PHQ-2) (18). Amongst PHQ-9 items, Q3-5 (trouble sleeping, low energy and appetite) enquire about somatic symptoms while Q6-9 items (feeling bad about yourself, trouble concentrating, moving or speaking slowly, suicidal ideation) assess non-somatic symptoms (19). EQ-5D-3L evaluates five health domains including mobility, self-care, usual activities, pain/discomfort and anxiety/depression (20), rated on three levels: 1 (no problem), 2 (some problems) to 3 (extreme problems). The traditional Chinese versions of PHQ-9 (6) and EQ-5D-3L (21) were used in this study.

All patients gave written informed consent for anonymized data to be analysed for publication and research purpose (22). The study was approved by the Chinese University of Hong Kong – New Territories East Cluster Clinical Research Ethics Committee.

### 2.4 Statistical analysis

We analysed patients enrolled in the HKDR who had completed both PHQ9 and EQ-5D-3L questionnaires since 13th March 2013 as part of a continuous quality improvement program. We censored these patients on the first CVD, CKD, death event or 31st December 2019, whichever came first. Longitudinal data on patient clinical outcomes were extracted from electronic patient medical records.

Continuous variables were expressed as mean ± standard deviation (SD), or median (inter-quartile range, IQR), and categorical variables, number (percentage). Between-group comparisons were analyzed by Chi-square test for categorical data, Wilcoxon two-sample test for continuous variables and Mann-Witney test for skewed data. Statistical analysis was performed using Statistical Package for Social Science (version 27.0). We compared the frequency of depression in patients with CVD (n=1436) or CKD (n=1181) at baseline. In the remaining patients with complete data (n=4429) for analysis, we used Kaplan–Meier estimator to demonstrate the survival probabilities of incident CVD and CKD and all-cause mortality in both depressed and non-depressed groups. Cox proportional hazards regression model was constructed to obtain hazard ratios (HRs, 95% confidence intervals [CI]) for: 1) all-cause mortality, 2) any incident CVD (stroke, PVD IHD), 3) IHD only, and 4) CKD, fitted to four models. Model 1 included age, gender, education, occupation, smoking status, duration of diabetes, body mass index (BMI), systolic and diastolic blood pressure, HbA1c and lipid profiles (LDL-C, HDL-C, triglycerides). Model 2 included variables in Model 1 plus Ln (urine ACR+1), eGFR, use of lipid-lowering drugs, angiotensin converting enzyme inhibitors (ACEI) or angiotensin II receptor blockers (ARB), other anti-hypertensive drugs and anti-diabetic drugs. Model 3 included variables in Model 2 plus frequency of self-reported adherence to balanced diet (never/no/occasional/yes), vigorous exercise corresponding to brisk walking > 30 minutes (no regular physical activity/1-3 times per week/3-4 times per week/5 times per week/>5 times per week), SMBG (yes/no), medication adherence level (1-100%) in the past 3 months and whether they had regular follow-up visits in past year. Model 4 included variables in Model 3 plus mean scores of EQ-5D-3L domains (excluding anxiety/depression) to adjust for baseline HRQoL. For CKD outcome, models 1 to 4 excluded Ln (urine ACR+1) and eGFR. In sensitivity analysis, we compared HRs of PHQ-2 versus PHQ-9 as well as independent risk associations of each item of PHQ-9 with clinical outcomes.

To examine the association between each individual item in the PHQ-9 questionnaire and all-cause mortality, all-CVD outcomes (including IHD, PVD and stroke), and CKD, hazard ratios were calculated and adjusted for all covariates in model 4. All regressions on each PHQ-9 item were adjusted for other PHQ-9 items.

In sensitivity analysis, we compared HRs of PHQ-2 versus PHQ-9 as well as independent risk associations of each item of PHQ-9 with clinical outcomes. This study followed the Strengthening the Reporting of Observational Studies in Epidemiology (STROBE) reporting guideline (23).

## 3 Results

### 3.1 Baseline characteristics

**Supplementary Figure 1** shows the flow chart of patient recruitment. Amongst 6818 Chinese patients with T2D enrolled in the HKDR with both PHQ-9 and EQ-5D-3L-5D data, 1436 patients had prior CVD (21%) and 1181 (17%) had CKD. In these patients with prior events, the prevalence of depression was 15.4% (15.6% for CVD and 17.0% for CKD). After excluding these patients with prior events, in the prospective cohort (n=4525), 537 patients (11.9%) had depressive symptoms based on PHQ-9≥7. Patients with depression were more likely to be women, younger, unemployed and had lower education attainment than the non-depressed group. They were less likely to perform regular exercise and more likely to forget or self-adjust medications. Patients with depression had higher serum triglyceride, HbA1c, ACR and eGFR, and were more likely to be treated with insulin. Overall, 5.33% of patients reported at least some problems in mobility, 1.46% for self-care, 4.66% for usual activities, 35.6% for pain/discomfort and 19.1% for anxiety/depression. The depressed group had more severe problems in all EQ-5D-3L domains (**Table 1**). For PHQ-9 items, 10% of patients reported somatic symptoms (Q3-5) for at least 7 days during the last 14 days, as compared to 5% for anhedonia (Q1), 3% for negative moods (Q2) and 3% for non-somatic (Q6-9) complaints (**Supplementary Table 2**). The majority of patients with T2D and co-morbid depression rated experiencing sleeping problems in Q3 (n= 330, 61.4%) and tired and lack of energy in Q4 (n = 299, 55.7%) on somatic complaints in more than half the days in the past 2 weeks.

**Table 1:** Baseline clinical profiles, patient reported outcomes and clinical events in Chinese patients with type 2 diabetes in the Hong Kong Diabetes Register (2013-2019) stratified by depression defined as PHQ-9 score ≥7.

	No depression (*n* = 3988)		Depression (*n* = 537)		P-value
	Mean or Frequency	SD or %	Mean or Frequency	SD or %	
Demographics					
**Age (years)**	55.9	10.6	54.4	10.6	**0.001**
**Gender (Male)**	2287	57.3%	259	47.3%	**<0.001**
**Occupation status**					**<0.001**
Employed	2215	55.5%	247	46.0%	
Unemployed	1770	44.4%	290	54.0%	
**Highest education attained**					**0.016**
Primary school, illiterate or others	913	22.9%	148	27.6%	
Middle school	1855	46.5%	259	48.2%	
Higher school	384	9.6%	44	8.2%	
College or above	815	20.4%	86	16.0%	
Clinical risk factors					
Duration of diabetes (years)	8.6	7.5	9.2	7.8	0.064
Body mass index (kg/m^2^)	26.2	4.74	26.5	4.9	0.172
Systolic blood pressure (mmHg)	130	17.1	130	18.6	0.940
Diastolic blood pressure (mmHg)	75.1	10.8	75.5	11.3	0.364
Self-care and patient-reported outcomes					
**Use of tobacco**					0.155
Non-smoker	2883	72.3%	394	73.4%	
Current smoker	472	11.8%	74	13.8%	
Ex-smoker	630	15.8%	68	12.7%	
Missing	3	0.1%	1	0.2%	
**Regular physical activity in last 3 months**					**<0.001**
No regular physical activity	1464	36.7%	265	49.3%	
1-3 times per week	829	20.8%	95	17.7%	
3-4 times per week	337	8.4%	41	7.6%	
5 times per week	175	4.4%	11	2.0%	
>5 times per week	1140	28.6%	124	23.1%	
Missing	3	0.1%	1	0.2%	
**Adherence to a balanced diet in last 3 months**					0.396
Never	85	2.1%	16	3.0%	
No	286	7.2%	48	8.9%	
Occasional	1670	41.9%	219	40.8%	
Yes	1936	48.5%	252	46.9%	
Missing	11	0.3%	2	0.4%	
**Self-monitoring of blood glucose**	2942	73.8%	400	74.5%	0.091
Missing	288	7.2%	50	9.3%	
**Having regular follow-up**	3820	95.8%	521	97.0%	0.383
Missing	1	0.0%	0	0%	
EQ-5D-3L domains^a^					
***Mobility***					**<0.001**
No problems in walking about	3785	96.3%	427	80.7%	
Some problems in walking about	137	3.5%	100	18.9%	
Confined to bed	2	0.1%	2	0.4%	
Missing	64	1.6%	8	1.5%	
***Self-care***					**<0.001**
No problems with self-care	3884	99.1%	498	94.1%	
Some problems washing or dressing myself	34	0.9%	28	5.3%	
Unable to wash or dress myself	2	0.1%	2	0.4%	
Missing	68	1.7%	8	1.5%	
***Usual activities***					**<0.001**
No problems with performing my usual activities	3812	97.1%	430	81.3%	
Some problems with performing my usual activities	109	2.8%	93	17.6%	
Unable to perform my usual activities	3	0.1%	6	1.1%	
Missing	64	1.6%	8	1.5%	
***Pain/discomfort***					**<0.001**
No pain or discomfort	2655	67.8%	180	34.0%	
Moderate pain or discomfort	1218	31.1%	310	58.6%	
Extreme pain or discomfort	44	1.1%	39	7.4%	
Missing	71	1.8%	8	1.5%	
***Anxiety/depression***					**<0.001**
Not anxious or depressed	3405	87.1%	169	32.0%	
Moderately anxious or depressed	498	12.7%	330	62.5%	
Extremely anxious or depressed	6	0.2%	29	5.5%	
Missing	79	2.0%	9	1.7%	
**Medication adherence**					**<0.001**
Self-rated medication adherence score (0-100%)	92.4	12.2	88.5	16.8	
Missing	218	5.5%	29	5.4%	
Use of medications					
Lipid lowering drugs	2272	57%	308	57.4%	0.866
Antihypertensive drugs including ACEIs or ARBs	2524	63.3%	351	65.4%	0.349
Insulin	1011	25.4%	183	34.1%	**<0.001**
Oral anti-diabetic drugs	3505	87.9%	477	88.8%	0.530
Laboratory results					
HbA1c (%)	7.6	1.5	7.9	1.8	**0.005**
LDL-cholesterol (mmol/L)	2.4	0.7	2.4	0.8	0.097
HDL-cholesterol (mmol/L)	1.3	0.4	1.3	0.4	0.237
Triglyceride (mmol/L)	1.6	1.1	1.7	1.7	**0.014**
Spot urine albumin creatinine ratio (mg/mmol) ^c^	1.2	0.5-3.9	1.5	0.6-6.5	**<0.001**
eGFR (mL/min, 1.73m^2^)^b^	92.7	14.7	94.1	15.6	**0.028**
Clinical outcomes					
Follow up duration ^c^	5.6	4.5-6.2	5.4	4.3-6.1	0.408
All-cause mortality	115	2.9%	26	4.8%	**0.014**
All cardiovascular outcomes	133	3.3%	31	5.8%	**0.005**
Ischaemic heart disease	75	1.9%	20	3.7%	**0.005**
Chronic kidney disease	459	11.5%	74	13.8%	0.125

a EQ5D-3L items were rated on the following scale: 1-no problem, 2-some problems to 3-extreme problems.

b eGFR = glomerular filtration rate from CKD-EPI equation.

c Median and Interquartile range.

ACEI, angiotensin converting enzyme inhibitor; ARB, angiotensin receptor blocker.

Bold values represent p<0.05.

**Figure 1:**
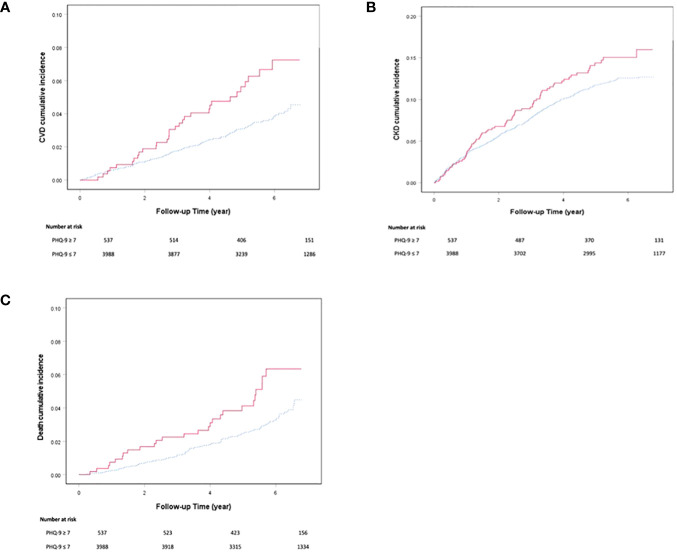
Cumulative incidence of **(A)** cardiovascular disease, **(B)** chronic kidney disease and **(C)** all-cause mortality derived from Kaplan-Meier analysis in Chinese patients with type 2 diabetes with or without depression defined by PHQ-9 score ≥ 7. The red solid line denotes patients with depression and the blue dotted line denotes patients without depression.

During a median follow-up period of 5.6 (IQR: 4.4-6.2) years, 141 patients (3.1%) died, 533 (11.8%) developed CKD and 164 (3.6%) developed CVD. In the latter group, 95 (57.9%) had IHD, 75 (45.7%) had other cardiovascular events (stroke: n=67, PVD: n= 8). The depressed group had a higher cumulative incidence of any CVD [5.8% versus 3.3%, p=0.005], IHD [3.7% versus 1.9%, p=0.005], all-cause mortality [4.8% versus 2.9% p=0.014] and CKD [13.8% versus 11.5%, p=0.125] than non-depressed group (**Figure 1**).

### 3.2 PHQ-9 items and clinical outcomes

Depression was significantly associated with increased CVD and IHD, where HR remained consistent in all 4 models after adjustment for demographics and clinical characteristics (model 1 – CVD: 1.97; 95%CI: 1.32–2.96, IHD: 2.41; 95%CI: 1.44-4.04), renal function (model 2– CVD: 1.86; 95%CI:1.24–2.80, IHD: 2.37; 95%CI: 1.41-3.99), medication use (model 3– CVD: 2.04; 95%CI: 1.32–3.16, IHD: 2.83; 95%CI: 1.62-4.94), and self-care (model 4– CVD: 1.99; 95%CI:1.25–3.18, IHD: 2.29; 95%CI: 1.25-4.21). Depression was associated with all-cause mortality in models 1-3 (model 1- 1.97; 95%CI: 1.27-3.06, model 2- 1.94; 95%CI: 1.24-3.03, model 3- 1.77; 95%CI: 1.09-2.88) but was rendered non-significant after adjusting for HRQoL (model 4- 1.54; 95%CI: 0.91-2.60), though HR still indicated same direction with important magnitude (**Table 2**).

**Table 2:** Incidence and hazard ratios (95% confidence interval) of depression defined by PHQ-9 score ≥7 for clinical outcomes in Chinese patients with type 2 diabetes.

Clinical outcomes	Depression (n = 537)	P-value
All-cause mortality (n=141)		
Unadjusted	1.75 (1.14-2.68)	**0.010**
Model 1	1.97 (1.27-3.06)	**0.003**
Model 2	1.94 (1.24-3.03)	**0.004**
Model 3	1.77 (1.09-2.88)	**0.022**
Model 4	1.54 (0.91-2.60)	0.108
All cardiovascular disease ^a^ (n=164)		
Unadjusted	1.80 (1.22-2.67)	**0.003**
Model 1	1.97 (1.32-2.96)	**0.001**
Model 2	1.86 (1.24-2.80)	**0.003**
Model 3	2.04 (1.32-3.16)	**0.001**
Model 4	1.99 (1.25-3.18)	**0.004**
Ischaemic heart disease (n=95)		
Unadjusted	2.06 (1.26-3.38)	**0.004**
Model 1	2.41 (1.44-4.04)	**<0.001**
Model 2	2.37 (1.41-3.99)	**0.001**
Model 3	2.83 (1.62-4.94)	**<0.001**
Model 4	2.29 (1.25-4.21)	**0.008**
Chronic kidney disease (n=533) ^b^		
Unadjusted	1.24 (0.97-1.58)	0.087
Model 1	1.24 (0.96-1.61)	0.100
Model 2	1.22 (0.95-1.58)	0.125
Model 3	1.14 (0.87-1.51)	0.344
Model 4	1.04 (0.77-1.40)	0.820

Model 1: Adjusted for age, gender, occupation status, highest education attained, smoking status, duration of diabetes, BMI, systolic and diastolic blood pressure, HbA1c, lipid profile (LDL cholesterol, HDL cholesterol, triglycerides).

Model 2: Model 1 + adjusted for Ln(ACR+1), eGFR, use of lipid lowering drugs, ACEI or ARB, other anti-hypertensive drugs, anti-diabetic drugs and insulin.

Model 3: Model 2 + adjusted for adherence to balanced diet, physical activity, level of medication adherence and self-monitoring of blood glucose in last 3 months and regular follow up in last 1 year.

Model 4: Model 3 + adjusted for ED-5D-3L Q1-Q4 (excluding Q5 on anxiety/depression)

a Including IHD, stroke and PVD.

b Models 1-4 for CKD outcome (eGFR<60 ml/min/1.73m^2^), Ln(urine ACR+1) and eGFR were excluded as covariates. Bold values represent p<0.05.

Detailed results of models 1 to 4 for all clinical outcomes (CVD, CKD, IHD and all-cause mortality) are shown in **Supplementary Tables 3**–**6**. In the final models, smoking, high HbA1c and lipid values were consistently associated with these adverse outcomes with physical activity and non-use of insulin associated with better outcomes. Although CKD was not associated with depression, patients who reported having regular exercise (3-4 times per week) had reduced risk of CKD (HR: 0.61, 95%CI: 0.41–0.89) (model 4). In the sensitivity analysis, HR of depression with all clinical outcomes were comparable using PHQ-2 (≥3) or PHQ-9 (≥7) scores to define depression (**Supplementary Tables 7**-**10**).

Amongst the nine items of PHQ-9 questionnaire, only Q4 (feeling tired or having little energy) was associated with all-cause mortality (HR:1.66, 95%CI: 1.30–2.12) after adjustment for covariates (**Table 3**).

**Table 3:** Hazard ratios (95% confidence interval) of questions from PHQ-9 for clinical outcomes in Chinese patients with type 2 diabetes.

PHQ9 Items	All-cause mortality	P-value	All CVD outcomes ^a^	p-value	CKD ^b^	P-value
Q1: Little interest or pleasure in doing things						
	0.79 (0.56-1.11)	0.165	1.31 (0.95-1.80)	0.100	1.11 (0.93-1.34)	0.254
Q2: Feeling down, depressed or hopeless						
	1.01 (0.63-1.61)	0.970	0.85 (0.54-1.34)	0.494	1.11 (0.85-1.44)	0.450
Q3: Trouble falling or staying asleep, or sleeping too much						
	0.95 (0.75-1.20)	0.678	1.17 (0.95-1.46)	0.147	1.03 (0.91-1.17)	0.611
Q4: Feeling tired or having little energy						
	1.66 (1.30-2.12)	**<0.001**	1.09 (0.84-1.42)	0.500	1.05 (0.90-1.22)	0.542
Q5: Poor appetite or overeating						
	1.05 (0.11-1.42)	0.771	0.99 (0.74-1.34)	0.954	1.02 (0.86-1.22)	0.824
Q6: Feeling bad about yourself — or that you are a failure or have let yourself or your family down						
	1.08 (0.73-1.60)	0.699	1.04 (0.71-1.53)	0.826	0.90 (0.71-1.14)	0.370
Q7: Trouble concentrating on things, such as reading the newspaper or watching television						
	0.94 (0.69-1.28)	0.696	0.99 (0.72-1.35)	0.935	1.07 (0.90-1.26)	0.436
Q8: Moving or speaking so slowly that other people could have noticed, or so fidgety or restless that you have been moving a lot more than usual						
	1.10 (0.77-1.57)	0.604	0.87 (0.56-1.33)	0.505	0.95 (0.74-1.21)	0.655
Q9: Thoughts that you would be better off dead, or thoughts of hurting yourself in some way						
	1.72 (0.92-3.23)	0.089	1.70 (0.91-3.17)	0.094	0.60 (0.33-1.10)	0.098

Adjusted for age, gender, occupation status, highest education attained, smoking status, duration of diabetes, BMI, systolic and diastolic blood pressure, HbA1c, lipid profile (LDL-cholesterol, HDL-cholesterol, triglycerides), Ln(ACR+1), eGFR, use of lipid lowering drugs, ACEI or ARB, other anti-hypertensive drugs, anti-diabetic drugs and insulin, adherence to balanced diet, physical activity, level of medication adherence and self-monitoring of blood glucose in last 3 months and regular follow up in last 1 year, ED-5D-3L: Q1-Q4 (excluding Q5 on anxiety/depression).

All regressions on each PHQ-9 item were adjusted for other PHQ-9 items.

a Including IHD, PVD and stroke.

b CKD outcome (eGFR<60 ml/min/1.73m^2^): Ln(urine ACR+1) and eGFR were excluded as covariates. Bold values represent p<0.05.

## 4 Discussion

Despite the growing burden of depression and T2D, their inter-relationships with PRO such as HRQoL and health behaviors on clinical outcomes had not been fully explored. In this ongoing clinic-based diabetes register set up for quality improvement purpose, 1 in 5 Chinese patients with T2D had either CVD or CKD at enrolment. Amongst these patients, 15% had depressive symptoms highlighting the importance of including PRO in patients with diabetes at high risk of multiple morbidities. In the remaining patients without complications, 11.9% had depression who were more likely to be women, had younger age and treated with insulin. They also had suboptimal control of risk factors, health behaviors and treatment adherence and worse HRQoL than those without depression.

Our results align with that reported in the UK and US diabetes population (24), which suggest a higher prevalence of depression among younger patients diagnosed with diabetes, particularly those with young-onset diabetes, compared to those with late-onset diabetes. Younger individuals with diabetes may face unique challenges and psychosocial burdens that contribute to a higher risk of depression in these regions. In contrast, depression prevalence increased with age in a study conducted in South India (25). This discrepancy could be attributed to region-specific trends and characteristics. In a separate study investigating age- and sex-specific hospital bed-day rates in a territory-wide cohort, we observed bimodal distribution associated with type 2 diabetes but not in those without. While the overall rate of hospital bed-days increased with age, among individuals diagnosed with T2D before the age of 40, 38.4% of hospital bed-days were attributed to mental health disorders (26). This highlights the severity of the issue and underscores the need for more comprehensive screening, interventions, and support services targeting mental health problems in young individuals with diabetes.

After 6 years of observations, patients with depression were 2 times more likely to develop CVD, mainly due to IHD, and all-cause death. These risk associations remained significant after adjusting for demographic and cardiometabolic risk factors, medications, self-care and HRQoL, albeit with some attenuation after adjusting for HRQoL. Exploratory analysis suggested that good self-care was associated with reduced risk of CKD. For the first time, we found that a single item Q4 (tiredness, low energy) in PHQ-9 was independently associated with all-cause mortality, suggesting that patients with failure to concentrate, excessive tiredness, motor retardation or restlessness required further evaluation of psychosocial-behavioural health. In support of professional practice guidelines (13), our results confirmed the importance of collecting PROs such as PHQ-9, EQ-5D-3L and psychosocial-behavioral factors for prognostication and providing holistic care to improve outcomes (1).

### 4.1 Associations of depression with CVD and all-cause mortality adjusting for HRQoL

In this study, young patients and women had higher prevalence of depression than their counterparts. Given the close associations between depression and clinical outcomes, our observations accorded with the higher incidence of all-cause and cardiovascular events in Asian women than men with diabetes (27). In agreement with other researchers, we also found that depression was associated with smoking, hypertension, poor metabolic control, albuminuria (28), suboptimal self-care (29) and drug non-adherence (30) which contributed to increased risk for CVD. In other studies, adherence to diet and exercise, SMBG (31) and foot care (32) were associated with reduced morbidity and mortality in patients with T2D. The introduction of risk assessment and education program at PWH had closed some care gaps as evidenced by similar use of ACEi/ARB and statin as well as similar frequency of SMBG between the depressed and non-depressed groups. However, depressed patients remained more likely to be treated with insulin, had higher HbA1c, worse lipid profiles, heavier albuminuria and reported poorer drug adherence and physical inactivity than the non-depressed group calling for more personalized treatment in these patients.

Compared with the non-depressed group, patients with depression were less likely to have balanced diet by 2.6% and regular exercise by 12.6%. In line with reports from European patients (33), after adjusting for these confounders including socioeconomic status represented by level of education and occupation, depression was associated with 2 times increased risk of CVD, notably IHD. These findings concurred with our previous report of 2 times increased risk of CVD in Chinese patients with T2D diagnosed with depression who received specialist care (12). In the current cohort, associations of depression based on PHQ-9 and CVD was attenuated after adjusting for HRQoL, albeit remained significant. Depression was also associated with 2 times increased risk of all-cause death, which was rendered non-significant after HRQoL adjustment.

On the other hand, we did not find an association between depression and CKD in both unadjusted and adjusted models. In another Chinese cohort, 37.8% had depression which was associated with CKD stages in a graded manner (34). Chinese adults with normal kidney function and severe depressive symptoms had 39% higher risk of rapid decline in kidney function than those without depression (35). Using PHQ-9≥10 to define depression, other researchers had reported an adjusted odds ratio of 1.36 (95% CI: 1.04-1.77) for microalbuminuria in patients with T2D (36). In this study, we excluded patients with CKD (eGFR<60 ml/min/1.73m^2^) at baseline, 17% of whom had depression. In the remaining patients without or with early CKD, longer follow-up will be needed to evaluate its association with depression and deterioration of renal function.

The magnitude of association between depression, CVD, IHD and all-cause mortality is consistent even after accounting for the influence of HRQoL and PROs, suggests that this relationship holds true across diverse patient populations with diabetes, regardless of their quality of life, lifestyle and self-management practices. These findings highlight the potential impact of mental health conditions on the development of cardiovascular complications in all patients with diabetes, denoting the importance to develop evidence-based policies and prevention interventions that address not only the acute health conditions of patients with diabetes, but also provide comprehensive support for their psychiatric well-being.

The complex nature of diabetes is evident, as studies have demonstrated strong intercorrelations and impacts among its physical, and psychosocial components (37, 38). This complements our previous study results which modelled that individuals diagnosed with T2D before the age of 40 may accrue an average of 100 inpatient bed days when they reach 75 years old, with approximately one-third of hospitalizations attributed to mental illness (39). Considering the 2023 pricing of public hospital psychiatric bed day cost at $300 USD in Hong Kong (40), and extrapolating the modelled results to the territory-wide cohort of 21,000 patients diagnosed with diabetes before the age of 40 (39), it is projected that an estimated total of $210 million USD may be spent on long-term mental illness-related hospitalizations in patients YOD over the next 35 years in Hong Kong. It is important to note that this estimation excludes potential costs associated with comorbid depression, such as cardiovascular disease (CVD) and mortality, in patients with diabetes. Another study conducted in Singapore identified one of the highest healthcare utilization clusters was characterized by a high prevalence of depression in women under the age of 65 with short-to-moderate disease duration (41). These findings emphasize the need for a holistic approach to diabetes management that takes into account the multifaceted aspects of the disease.

### 4.2 Association of PHQ-9 Q4 (lack of energy) with all-cause mortality in patients with T2D

In this cohort, 11.9% of patients had depressive symptoms and the majority had not been diagnosed. Depressive symptomatology can be heterogeneous with diverse cultural norms, perceptions and interpretations. International practice guidelines suggested screening for depression in patients with diabetes, especially in those with poor glycaemic control (13). However, in busy clinic settings, routine administration of PHQ-9 could be challenging, calling for a simple but robust screening tool to identify patients with comorbid T2D and depression.

In Hong Kong, we reported higher discriminatory power with PHQ-9 than Center for Epidemiological Studies Depression (CES-D) scale for depression screening (19). The latter puts more emphasis on the affective component of depression. The optimal cutoff value to detect depressive symptoms varied between populations and settings. For example, the optimal cut-off score for PHQ-9 in outpatient population with diabetes was 9 in Malawi (sensitivity: 64%, specificity: 94%) (42), 12 in Netherlands (sensitivity: 75.7%, specificity: 80.0%) (43) and 7 in Poland (sensitivity 90.62%; specificity 90.22%) (44). Before we introduced PHQ9 in our routine service, 99 randomly selected patients enrolled in the register were interviewed by psychiatrists using the Mini International Neuropsychiatric Interview as the golden standard (19). Utilizing receiver operating characteristic (ROC) analysis, the area under the curve (AUC) was 0.85(95%CI:0.76–0.94) with a cutoff score of ≥7 yielding an optimal balance between sensitivity (82.6%) and specificity (73.7%). By contrast the widely accepted score of 10 had comparable specificity (84.2%) but poor sensitivity (56.5%). When the categorical algorithm was used to define major depressive disorder based on 1) 5 of 9 items including item 1 (anhedonia) or 2 (depressed mood) being endorsed as “more than half the days” or “nearly every day” or 2) Item 9 (suicidal ideation) regardless of duration, the sensitivity was 39.1% and specificity, 96.1% (19).

In this study, Chinese patients with T2D were more likely to report somatic symptoms with Q4 in PHQ-9 (feeling tired or having little energy) being independently associated with 57% increased risk of all-cause mortality. Other researchers had reported a correlation coefficient of 0.50 between Q4 and four items in Fatigue Questionnaire (45). Patients with diabetes who reported fatigue were 10.37 times more likely to have depression than those without symptoms of fatigue (46, 47). The robust associations of all-cause mortality with Q4 of PHQ-9, at least in Chinese patients with T2D, called for routine enquiry of physical activity and energy level to identify patients at risk of depression. Apart from using the overall PHQ-9 score to screen for depression, a high score for Q4 should alert healthcare providers to conduct comprehensive assessment of mental health for psychosocial interventions.

In a recent network analysis study conducted in Canada, involving 1,796 middle-aged patients with diabetes (48), findings indicate that early targeted intervention on behavioral activation and cognitive restructuring that address “failure” (item 6 in PHQ-9),”uncontrollable worry, “excessive worrying” and “difficulty relaxing” [item 2-4 in Diabetes Distress Scale (DSS-17)] may potentially prevent the development of future comorbid mental conditions in individuals with type 2 diabetes (48). In our current study, we reported that the majority of our patients with T2D and comorbid depression (PHQ-9 ≥7) scored high on somatic problems such as “sleeping difficulties” (item 3) and “lack of energy” (item 4) in PHQ-9. The inclusion of network analysis in future investigations on depression in Chinese patients with diabetes holds significant potential to contribute valuable insights into the complex dynamics and interactions among symptoms and domains of depression. This approach has the capacity to enhance our ability to precisely identify and characterize different subtypes of depression in this population for designing effective targeted interventions.

### 4.3 Study implications

The myriad of complications associated with diabetes, use of long-term medication, necessity for regular follow-up visits, and demand for lifestyle changes may adversely impact an individual’s lifestyles, perspectives and emotions. These factors can be modified by sociodemographic factors such as education, poverty and personal relationships in family or work. All these dimensions can interact in a complex manner to influence quality of life which in turn can feed back on these psychosocial-behavioral dimensions. Apart from influencing self-care, these perceptions and emotions may be associated with biological changes (8). There are now growing interests on the associations of gene-environment interactions with depression and health behaviors in diabetes with inconclusive results (49). In a recent Chinese study, dietary intake, alcohol drinking and smoking, physical activity, and socioeconomic status were reported to interact with genetic variants to modulate the risks of impaired fasting glucose and impaired glucose tolerance (50). In this light, despite the many technological advances in the field of diabetes, such as medications and monitoring tools, there remain considerable care gaps with high complications rates calling for better understanding of genetic factors and PROs and clinical outcomes to improve physical, mental and behavioral health (1, 51).

Dysregulation of neurohormonal and immune systems may underlie the clustering of subphenotypes including cardiovascular-renal complications (52). In randomized controlled trials, multicomponent care including use of medications and anti-depressants, lifestyle modification and psychosocial support improved depression, PRO and cardiometabolic risk factors in patients with T2D and depression (53). In a secondary analysis of the Look Ahead Study, obese patients with T2D receiving intensive lifestyle intervention had reduced incidence of depression and CKD than the control group (54, 55). Our group also reported benefits of peer support using telephone counselling in reducing hospitalizations in patients with T2D especially in those with negative emotions (56). Other community- and family-based interventions including use of lifestyle intervention and digital technologies also improved QoL in patients with diabetes (57). Taken together, there is a need to integrate PHQ-9 questionnaire into routine diabetes screening and assessment to detect these high risk individuals early for personalized care in order to improve their physical, social and mental health.

## 5 Strengths and limitations

The comprehensiveness of data collection including biomedical-psychosocial-behavioural factors, HRQoL and clinical outcomes is a major strength of the study, albeit not without limitations. Our cohort was recruited in an ambulatory clinic setting catering patients with more complex and specialized healthcare needs, thus may restrict the broader applicability of our findings to a primary care context. Furthermore, in this quality improvement program, patients with PHQ-9 score >10 were referred to an on-call endocrinologist and psychiatrist while patients with score of 9-10 received counselling from trained nurses. The absence of a comprehensive psychiatric evaluation for all patients may limit our ability to accurately determine the true prevalence of depression in this population. Health behaviors such as adherence to medication, diet and physical activity in last 3 months were subject to recall bias. Despite their frequent coexistence, diabetes and depression can independently have negative impacts on clinical outcomes. In this diabetes register, we did not have patients without T2D and could not test the mediation effects of depression-alone, T2D-alone and co-morbid depression and T2D on clinical outcomes compared to those with neither condition.

It is important to note that our study is designed to demonstrate the risk associations between clinical outcomes and baseline depression and PROs. Therefore, conclusions cannot be drawn regarding the temporal changes of depression and PROs over time. Furthermore, we acknowledge that as many as 64% of patients with depression might have comorbid mental disorders (58) which could confound our results. However, due to the pragmatic nature of the register, we did not capture full details of other mental illness in these patients. In this context, randomized controlled trials had confirmed the benefits of multidisciplinary care on physical and mental health in patients with T2D and depression (53), in support of identifying these patients early for intervention. The attenuation of risk association between depression and clinical outcomes by HRQoL suggested that other social, environmental and behavioral factors might be important which calls for more systemic data collection to inform interventions beyond healthcare. To unravel these complex inter-relationships, advanced methodology such as structural equation modelling will be needed to quantify the causal effects of these factors to inform practice and policies (59).

## 6 Conclusions

In conclusion, the association between depression, CVD and IHD remains significant across all patients with diabetes, regardless of their HRQoL and health behaviours. Despite the association between depression and all-cause mortality being attenuated after adjusting for HRQoL, the effect size and direction of association remained substantial. Our findings highlight the importance of holistic diabetes management with comprehensive support for mental well-being. Given the complex nature of diabetes, including PROs such as PHQ-9, EQ-5D-3L and health behaviors can further increase the value of a regular structured assessment program for identifying high risk patients for holistic management. In Chinese patients with T2D and depression, somatic complaints were common with lack of energy captured by item 4 in PHQ-9 being independently associated with all-cause mortality. In busy clinic settings, patients who reported physical inactivity or low energy level warrant further evaluation of emotional health for early intervention.

## Data availability statement

The datasets presented in this article are not readily available because Due to legal restrictions, patient-level data cannot be made publicly available. Aggregate data may be available upon reasonable request. Requests to access the datasets should be directed to jchan@cuhk.edu.hk.

## Ethics statement

This study involving humans was approved by The Chinese University of Hong Kong – New Territories East Cluster Clinical Research Ethics Committe. This study was conducted in accordance with the local legislation and institutional requirements. Written informed consent for participation in this study was provided by the participants.

## Author contributions

Y-LY: Data curation, Formal analysis, Methodology, Writing – original draft, Writing – review & editing, Conceptualization, Investigation, Project administration, Visualization. K-LL: Conceptualization, Data curation, Formal analysis, Investigation, Methodology, Project administration, Visualization, Writing – original draft, Writing – review & editing. EL: Conceptualization, Data curation, Formal analysis, Investigation, Methodology, Project administration, Supervision, Visualization, Writing – original draft, Writing – review & editing. T-FY: Conceptualization, Data curation, Formal analysis, Investigation, Methodology, Project administration, Visualization, Writing – original draft, Writing – review & editing. AY: Data curation, Investigation, Project administration, Supervision, Visualization, Writing – review & editing. HW: Data curation, Investigation, Project administration, Supervision, Visualization, Writing – review & editing, Validation. KW: Methodology, Supervision, Writing – review & editing, Project administration, Validation. AK: Data curation, Project administration, Supervision, Writing – review & editing. EC: Data curation, Supervision, Writing – review & editing, Project administration. RM: Data curation, Project administration, Supervision, Writing – review & editing. TY: Data curation, Project administration, Writing – review & editing. K-ML: Data curation, Project administration, Writing – review & editing. RO: Data curation, Project administration, Writing – review & editing. AL: Data curation, Project administration, Supervision, Writing – review & editing. JL: Data curation, Formal analysis, Methodology, Project administration, Supervision, Visualization, Writing – original draft, Writing – review & editing. JC: Conceptualization, Data curation, Formal analysis, Investigation, Methodology, Project administration, Resources, Supervision, Visualization, Writing – review & editing.
