# Supplementary material for: Associations of comorbid depression with cardiovascular-renal events and all-cause mortality accounting for patient reported outcomes in individuals with type 2 diabetes: a 6-year prospective analysis of the Hong Kong Diabetes Register
Source: Front Endocrinol (Lausanne). 2024 Mar 22;15:1284799. doi: 10.3389/fendo.2024.1284799 (PMC10999250; doi:10.3389/fendo.2024.1284799)
Supplement: Supplementary file 1 [file DataSheet_1.pdf]

Associations of comorbid depression with cardiovascular-renal events and all-cause mortality accounting for patient reported outcomes in individuals with type 2 diabetes: A 6-year prospective analysis of the Hong Kong Diabetes Register

Table of Contents

Supplementary table 1. Definitions of clinical outcomes by International Classification of Diseases (ICD-9/ICD-10) 2

Supplementary figure 1. Study flow chart 3

Supplementary table 2 Distribution of Items of PHQ-9 in Chinese patients with type 2 diabetes (N=4525). 5

Supplementary table 3. Stepwise logistic regression for all-cause mortality in Chinese patients with T2D (n = 141) 7

Supplementary table 4. Stepwise logistic regression for all CVD outcomes (including IHD) in Chinese patients with T2D (*n* = 164) 10

Supplementary table 5. Stepwise logistic regression for IHD in Chinese patients with T2D (*n* = 95) 10

Supplementary table 6. Stepwise logistic regression for CKD in Chinese patients with T2D (*n* = 533) 13

Supplementary table 7. Logistic regression for all-cause mortality in Chinese patients with type 2 diabetes, using PHQ-2 ≥3 as cut-off (*n* = 141) 16

Supplementary table 8. Logistic regression for all CVD outcomes (including IHD) in Chinese patients with type 2 diabetes, using PHQ-2≥3 as cut-off (*n* = 164). 19

Supplementary table 9. Logistic regression for IHD in Chinese patients with type 2 diabetes, using PHQ-2 ≥3 as cut-off (*n* = 95) 22

Supplementary table 10. Logistic regression for CKD in Chinese patients with type 2 diabetes, using PHQ-2 ≥ 3 as cut-off (*n* = 533) 24

Supplementary table 1. Definitions of clinical outcomes by International Classification of Diseases (ICD-9/ICD-10)

Clinical outcomes	ICD-9/ICD-10 definition
**Cardiovascular disease (CVD)**	ICD-9 code: Acute myocardial infarction (410), other acute and subacute forms of ischemic heart disease (411), old myocardial infarction (412), angina pectoris (413), other forms of chronic ischemic heart disease (414); subarachnoid haemorrhage (430), intracerebral haemorrhage (431), other and unspecified intracranial haemorrhage (432), occlusion and stenosis of precerebral arteries (433), occlusion of cerebral arteries (434), acute, but ill-defined, cerebrovascular disease (436), other and ill-defined cerebrovascular disease (437), late effects of cerebrovascular disease (438); amputation of lower limb (procedure code of 84.1) but discharges with a traumatic amputation diagnosis code (895-897) were excluded, diabetes with peripheral circulatory disorders (250.7), gangrene (785.4), peripheral angiopathy in diseases classified elsewhere (443.81), peripheral vascular disease, unspecified (443.9), other (peripheral) vascular shunt or bypass (procedure code 39.29); insertion of non-drug-eluting peripheral vessel stent(s) (procedure code 39.90); other procedure codes (surgical revascularization: 38.08, 38.18, 38.38, 38.48, 38.68, 38.88, 39.25, 39.49, 39.56, 39.57, 39.58, 39.59, 39.99; endovascular revascularization: 00.55, 17.56, 39.50, 39.79)
**Ischemic heart disease (IHD)**	ICD-9 code: Acute myocardial infarction (410), other acute and subacute forms of ischemic heart disease (411), old myocardial infarction (412), angina pectoris (413), other forms of chronic ischemic heart disease (414)
**Chronic Kidney Disease (CKD)**	eGFR<60, exclude acute kidney failure (584): for new event, 2 eGFR<60 separated by 90-365 days, for history, the eGFR at baseline or the latest eGFR before baseline <60; haemodialysis dialysis (procedure code 39.95) with diagnosis of chronic kidney disease (585) or renal failure (586), peritoneal dialysis (procedure code 54.98),transplant of kidney (procedure code 55.6), complications of transplanted kidney (996.81), persons with a condition influencing their health status; organ or tissue replaced by transplant; kidney (V42.0)
**All-cause mortality**	ICD-10 cause of death from Hong Kong Death Register

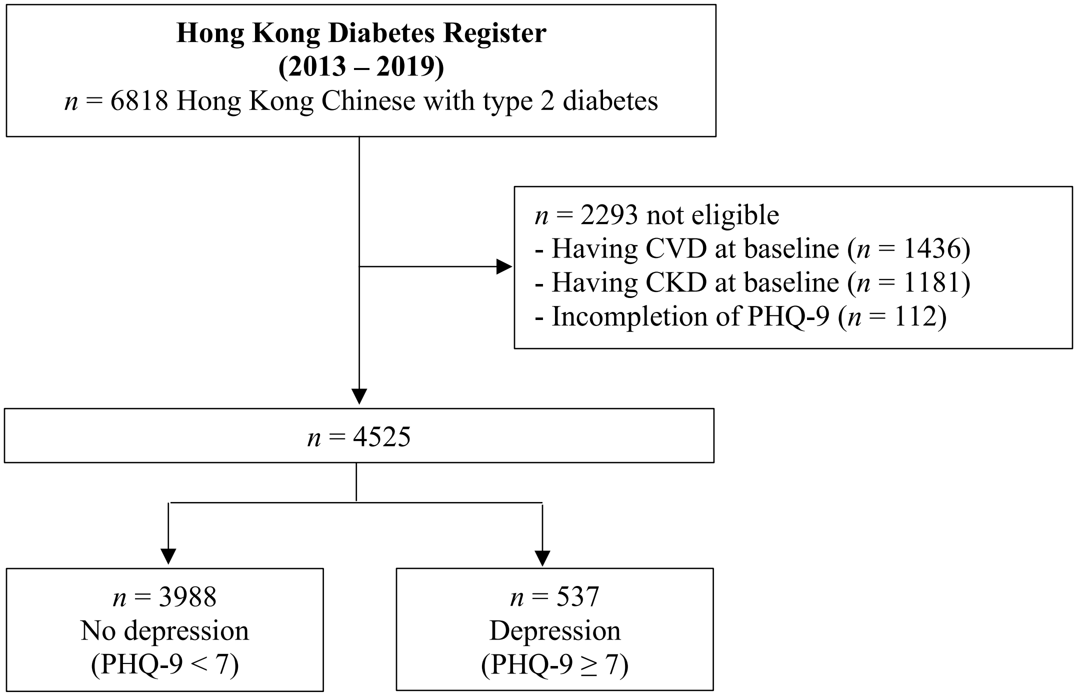

Supplementary figure 1. Study flow chart

Supplementary table 2 Distribution of Items of PHQ-9 in Chinese patients with type 2 diabetes (N=4525).

PHQ-9 Items[a]	Item description	No depression PHQ-9<7 (n=3988)	Depression PHQ-9≥7 (n=537)	P-value
**Overall score**	-	1.72 (1.82)	10.22 (3.92)	**<0.01**
Q1: Little interest or pleasure in doing things	Anhedonia			**<0.01**
Not at all		3164 (79.3%)	66 (12.3%)	
Several days		773 (19.4%)	279 (52.0%)	
More than half the days		46 (1.2%)	127 (23.6%)	
Nearly every day		5 (0.1%)	65 (12.1%)	
Q2: Feeling down, depressed or hopeless	Depressed mood			**<0.01**
Not at all		3686 (92.4%)	174 (32.4%)	
Several days		287 (7.2%)	254 (47.3%)	
More than half the days		14 (0.4%)	80 (14.9%)	
Nearly every day		1 (0.0%)	29 (5.4%)	
Q3: Trouble falling or staying asleep, or sleeping too much	Somatic symptoms			**<0.01**
Not at all		2447 (61.4%)	47 (8.8%)	
Several days		1219 (30.6%)	160 (29.8%)	
More than half the days		227 (5.7%)	165 (30.7%)	
Nearly every day		95 (2.4%)	165 (30.7%)	
Q4: Feeling tired or having little energy?	Somatic symptoms			**<0.01**
Not at all		2402 (60.2%)	25 (4.7%)	
Several days		1383 (34.7%)	213 (39.7%)	
More than half the days		164 (4.1%)	183 (34.1%)	
Nearly every day		39 (1.0%)	116 (21.6%)	
Q5: Poor appetite or overeating?	Somatic symptoms			**<0.01**
Not at all		3159 (79.2%)	116 (21.6%)	
Several days		771 (19.3%)	228 (42.5%)	
More than half the days		55 (1.4%)	128 (23.8%)	
Nearly every day		3 (0.1%)	65 (12.1%)	
Q6: Feeling bad about yourself — or that you are a failure or have let yourself or your family down?	Non-somatic symptoms			**<0.01**
Not at all		3667 (92.0%)	172 (32.0%)	

Several days		294 (7.4%)	225 (41.9%)	
More than half the days		22 (0.6%)	98 (18.2%)	
Nearly every day		5 (0.1%)	42 (7.8%)	
Q7: Trouble concentrating on things, such as reading the newspaper or watching television?	Non-somatic symptoms			**<0.01**
Not at all		3597 (90.2%)	161 (30.0%)	
Several days		341 (8.6%)	231 (43.0%)	
More than half the days		37 (0.9%)	90 (16.7%)	
Nearly every day		13 (0.3%)	55 (10.2%)	
Q8: Moving or speaking so slowly that other people could have noticed? Or so fidgety or restless that you have been moving a lot more than usual?	Non-somatic symptoms			**<0.01**
Not at all		3832 (96.0%)	249 (46.4%)	
Several days		138 (3.5%)	193 (35.9%)	
More than half the days		16 (0.4%)	64 (11.9%)	
Nearly every day		2 (0.1%)	31 (5.8%)	
Q9: Thoughts that you would be better off dead, or thoughts of hurting yourself in some way?	Non-somatic symptoms			**<0.01**
Not at all		3973 (99.6%)	439 (81.8%)	
Several days		14 (0.4%)	72 (13.4%)	
More than half the days		0 (0%)	21 (3.9%)	
Nearly every day		1 (0.0%)	5 (0.9%)	

[a] *PHQ-9 items were rated on the following scale: 0 – Not at all, 1- Several days, 2- More than half the days, 3- Nearly every day*
Bold values represent p<0.05

Supplementary table 3. Stepwise logistic regression for all-cause mortality in Chinese patients with T2D (n = 141)

	Model 1		Model 2		Model 3		Model 4	
	HR (95% Cl)	P-value	HR (95% Cl)	P-value	HR (95% Cl)	P-value	HR (95% Cl)	P-value
**PHQ-9 score ≥ 7**	1.97 (1.27-3.06)	**0.003**	1.94 (1.24-3.03)	**0.004**	1.79 (1.09-2.91)	**0.020**	1.54 (0.91-2.60)	0.108
**Demographics**								
Age (years)	1.07 (1.05-1.10)	**<0.001**	1.09 (1.06-1.12)	**<0.001**	1.08 (1.05-1.11)	**<0.001**	1.08 (1.05-1.11)	**<0.001**
Male gender (ref: female)	1.22 (0.75-1.96)	0.422	1.22 (0.76-1.98)	0.414	1.08 (0.65-1.82)	0.763	1.13 (0.67-1.90)	0.656
Employed (ref: unemployed)	0.74 (0.48-1.13)	0.163	0.76 (0.50-1.15)	0.192	0.62 (0.39-0.99)	0.045	0.63 (0.40-1.01)	0.057
Highest education attained (ref: primary, illiterate and others)								
Middle school	1.09 (0.72-1.64)	0.689	1.09 (0.72-1.64)	0.692	1.12 (0.73-1.74)	0.601	1.12 (0.72-1.73)	0.626
Higher school	1.20 (0.62-2.31)	0.588	1.18 (0.61-2.27)	0.627	1.15 (0.57-2.35)	0.699	1.15 (0.56-2.34)	0.709
College or above	1.15 (0.65-2.02)	0.636	1.15 (0.65-2.03)	0.628	1.18 (0.64-2.18)	0.591	1.18 (0.64-2.19)	0.588
Smoking status (ref: non-smoker)								
Ex-smoker	2.13 (1.35-3.34)	**0.001**	2.16 (1.37-3.40)	**<0.001**	2.58 (1.58-4.22)	**<0.001**	2.61 (1.60-4.28)	**<0.001**
Current Smoker	1.98 (1.13-3.47)	**0.017**	1.88 (1.07-3.32)	**0.029**	2.17 (1.17-4.05)	**0.014**	2.12 (1.14-3.95)	**0.018**
Duration of diabetes (years)	1.00 (0.98-1.03)	0.694	1.01 (0.99-1.03)	0.452	1.01 (0.99-1.04)	0.324	1.01 (0.99-1.04)	0.348
**Clinical parameters**								
Body mass index	0.97 (0.92-1.01)	0.156	0.97 (0.93-1.02)	0.225	0.98 (0.93-1.03)	0.411	0.98 (0.93-1.03)	0.374
Systolic blood pressure	1.01 (1.00-1.02)	0.060	1.01 (1.00-1.02)	0.182	1.01 (1.00-1.02)	0.129	1.01 (1.00-1.02)	0.181
Diastolic blood pressure	1.00 (0.98-1.02)	0.916	1.00 (0.98-1.02)	0.935	1.00 (0.98-1.02)	0.870	1.00 (0.98-1.02)	0.951
HbA1c	1.28 (1.16-1.40)	**<0.001**	1.23 (1.11-1.36)	**<0.001**	1.27 (1.14-1.41)	**<0.001**	1.29 (1.15-1.43)	**<0.001**
LDL-cholesterol	0.88 (0.68-1.12)	0.293	0.80 (0.61-1.03)	0.084	0.82 (0.62-1.09)	0.171	0.81 (0.61-1.08)	0.147
HDL-cholesterol	0.95 (0.57-1.58)	0.831	0.87 (0.52-1.43)	0.575	0.90 (0.53-1.53)	0.695	0.91 (0.54-1.54)	0.725

Triglyceride	0.82 (0.62-1.09)	0.175	0.80 (0.60-1.06)	0.118	0.81 (0.60-1.11)	0.187	0.81 (0.60-1.10)	0.177
Ln (urine ACR + 1)			**1.26 (1.10-1.44)**	**<0.001**	**1.22 (1.05-1.41)**	**0.009**	**1.21 (1.05-1.40)**	**0.009**
Estimated GFR			1.01 (1.00-1.03)	0.142	1.02 (1.00-1.03)	0.082	1.02 (1.00-1.04)	0.068
**Drug use vs not on drug at baseline**								
Lipid lowering drugs			0.74 (0.51-1.08)	0.113	0.75 (0.51-1.12)	0.164	0.77 (0.52-1.15)	0.199
Antihypertensive drugs, including ACEIs or ARBs			0.87 (0.56-1.35)	0.536	0.96 (0.59-1.57)	0.884	0.97 (0.60-1.59)	0.910
Oral anti-diabetic drugs			**0.59 (0.35-0.98)**	**0.040**	**0.49 (0.27-0.88)**	**0.018**	**0.49 (0.27-0.89)**	**0.018**
Insulin			0.96 (0.63-1.49)	0.870	0.88 (0.54-1.41)	0.587	0.85 (0.53-1.37)	0.511
**Diabetes self-care**								
Self reported level of medication adherence (0-100)					1.00 (0.98-1.01)	0.633	1.00 (0.98-1.01)	0.689
Regular physical activity in last 3 months (ref: no regular physical activity)								
1-3 times per week					0.88 (0.49-1.60)	0.677	0.90 (0.49-1.64)	0.729
3-4 times per week					0.73 (0.35-1.52)	0.403	0.76 (0.36-1.59)	0.460
5 times per week					1.25 (0.52-3.02)	0.621	1.28 (0.53-3.11)	0.585
> 5 times per week					0.83 (0.52-1.31)	0.415	0.85 (0.54-1.35)	0.496
Adherence to a balanced diet in last 3 months (ref: never)								
No					1.45 (0.40-5.22)	0.571	1.39 (0.39-5.01)	0.616
Occasional					0.82 (0.25-2.68)	0.736	0.79 (0.24-2.59)	0.691
Yes					1.03 (0.32-3.38)	0.956	1.00 (0.31-3.27)	1.000
Self-monitoring of glucose (ref: none)					0.82 (0.51-1.33)	0.428	0.79 (0.49-1.29)	0.349
Regular follow up (ref: none)					1.57 (0.38-6.57)	0.535	1.63 (0.39-6.87)	0.504
**EQ5D-3L domains**								
Mobility							0.81 (0.38-1.73)	0.591
Self-care							1.46 (0.59-3.58)	0.412
Usual activities							1.50 (0.75-3.00)	0.257
Pain/discomfort							1.21 (0.84-1.73)	0.310

Note:

Model 1: Adjusted for age, gender, occupation status, highest education attained, smoking status, duration of diabetes, BMI, systolic and diastolic blood pressure, HbA1c, lipid profile (LDL cholesterol, HDL cholesterol, triglycerides).
Model 2: Model 1 + adjusted for Ln(ACR+1) , eGFR, use of lipid lowering drugs, ACEI or ARB, other anti-hypertensive drugs, anti-diabetic drugs, insulin
Model 3: Model 2 + adjusted for adherence to balanced diet, physical activity, level of medication adherence and self-monitoring of blood glucose in last 3 months and regular follow up in last 1 year.
Model 4: Model 3 + adjusted for ED-5D-3L Q1-Q4 (excluding Q5 on anxiety/ depression)
[a] Including IHD
[b] Models 1-4 for CKD outcome excluded adjustment for Ln(urine ACR+1) and eGFR
Bold values represent $p<0.05$

Supplementary table 4. Stepwise logistic regression for all CVD outcomes (including IHD) in Chinese patients with T2D (*n* = 164)

Supplementary table 5. Stepwise logistic regression for IHD in Chinese patients with T2D (*n* = 95)

	Model 1		Model 2		Model 3		Model 4	
	HR (95% CI)	P-value	HR (95% CI)	P-value	HR (95% CI)	P-value	HR (95% CI)	P-value
PHQ-9 score ≥ 7	2.43 (1.45-4.07)	**<0.001**	2.38 (1.41-4.01)	**0.001**	2.83 (1.62-4.94)	**<0.001**	2.29 (1.25-4.21)	**0.008**
**Demographics**								
Age (years)	1.04 (1.01-1.07)	**0.010**	1.04 (1.00-1.07)	**0.031**	1.04 (1.00-1.08)	0.058	1.03 (0.99-1.07)	0.095
Male gender (ref: female)	2.53 (1.37-4.65)	**0.003**	2.51 (1.35-4.69)	**0.004**	3.24 (1.60-6.58)	**0.001**	3.43 (1.67-7.05)	**<0.001**
Employed (ref: unemployed)	1.26 (0.77-2.06)	0.364	1.28 (0.78-2.11)	0.332	1.05 (0.61-1.82)	0.850	1.14 (0.65-1.99)	0.645
Highest education attained (ref: primary, illiterate and others)								
Middle school	1.56 (0.89-2.78)	0.123	1.57 (0.88-2.79)	0.125	1.67 (0.89-3.12)	0.110	1.66 (0.88-3.14)	0.118
Higher school	1.60 (0.70-3.68)	0.270	1.63 (0.71-3.76)	0.248	1.97 (0.83-4.68)	0.124	2.02 (0.85-4.81)	0.113
College or above	1.43 (0.69-2.97)	0.332	1.50 (0.72-3.10)	0.278	1.55 (0.69-3.45)	0.288	1.61 (0.72-3.61)	0.250
Smoking status (ref: non-smoker)								
Ex-smoker	1.17 (0.66-2.07)	0.595	1.19 (0.67-2.12)	0.551	1.51 (0.83-2.76)	0.180	1.49 (0.80-2.77)	0.205
Current smoker	2.15 (1.22-3.78)	**0.008**	2.20 (1.24-3.88)	**0.007**	2.63 (1.41-4.89)	**0.002**	2.67 (1.43-4.99)	**0.002**
Duration of diabetes (years)	1.03 (1.01-1.06)	**0.021**	1.03 (1.00-1.06)	**0.066**	1.04 (1.00-1.07)	**0.038**	1.04 (1.00-1.07)	**0.035**
**Clinical parameters**								
Body mass index (kg/m^2)	1.02 (0.97-1.07)	0.525	1.01 (0.96-1.07)	0.594	1.00 (0.94-1.06)	0.996	1.00 (0.94-1.06)	0.959
Systolic blood pressure (mmHg)	1.01 (1.00-1.03)	0.110	1.01 (0.99-1.03)	0.247	1.01 (1.00-1.03)	0.113	1.01 (1.00-1.03)	0.134
Diastolic blood pressure (mmHg)	0.99 (0.96-1.02)	0.373	0.99 (0.96-1.01)	0.337	0.98 (0.95-1.01)	0.236	0.98 (0.95-1.01)	0.250
HbA1c (%)	1.01 (0.87-1.16)	0.935	0.98 (0.84-1.14)	0.778	1.00 (0.84-1.17)	0.959	0.98 (0.82-1.16)	0.810
LDL-cholesterol (mmol/L)	1.36 (1.03-1.80)	**0.028**	1.32 (0.99-1.76)	0.062	1.41 (1.03-1.93)	**0.032**	1.46 (1.07-1.99)	**0.018**
HDL-cholesterol (mmol/L)	0.97 (0.50-1.89)	0.928	1.00 (0.51-1.95)	0.996	1.04 (0.51-2.13)	0.912	1.03 (0.50-2.11)	0.936
Triglyceride (mmol/L)	1.15 (0.94-1.39)	0.174	1.13 (0.91-1.40)	0.267	1.21 (0.94-1.56)	0.131	1.22 (0.96-1.55)	0.113
Ln(urine ACR + 1)			1.15 (0.98-1.36)	0.092	1.17 (0.98-1.40)	0.093	1.15 (0.95-1.37)	0.147
eGFR (mL/min/1.73 m^2)			0.99 (0.97-1.01)	0.287	1.00 (0.98-1.02)	0.732	1.00 (0.98-1.02)	0.691
**Drug use at enrolment (ref: not on respective drug)**								

Lipid lowering drugs			1.05 (0.66-1.66)	0.839	1.08 (0.66-1.78)	0.757	1.13 (0.68-1.87)	0.640
Antihypertensive drugs, including ACEIs or ARBs			0.78 (0.46-1.33)	0.367	0.88 (0.49-1.58)	0.664	0.94 (0.51-1.72)	0.840
Oral anti-diabetic drugs			1.18 (0.55-2.54)	0.673	1.23 (0.43-3.50)	0.698	1.76 (0.53-5.83)	0.357
Insulin			1.03 (0.60-1.79)	0.910	1.03 (0.57-1.87)	0.911	1.04 (0.58-1.88)	0.891
**Diabetes self-care**								
Self-reported level of medication adherence (0-100)					1.00 (0.98-1.02)	0.907	1.00 (0.98-1.02)	0.934
Regular physical activity in last 3 months (ref: no regular physical activity)								
1-3 times per week					1.54 (0.84-2.81)	0.163	1.64 (0.89-3.03)	0.116
3-4 times per week					0.40 (0.12-1.35)	0.142	0.43 (0.13-1.47)	0.178
5 times per week					0.39 (0.05-2.86)	0.351	0.41 (0.06-3.09)	0.390
> 5 times per week					0.95 (0.53-1.72)	0.877	0.99 (0.54-1.81)	0.967
Adherence to a balanced diet in last 3 months (ref: never)								
No					1.00 (0.11-8.74)	0.996	0.93 (0.11-8.23)	0.950
Occasional					1.59 (0.22-11.85)	0.649	1.50 (0.20-11.2)	0.690
Yes					1.47 (0.20-11.00)	0.705	1.42 (0.19-10.6)	0.731
Self-monitoring of glucose (ref: none)					0.98 (0.51-1.86)	0.940	1.00 (0.51-1.94)	0.996
Regular follow up (ref: none)					0.46 (0.14-1.51)	0.199	0.44 (0.13-1.45)	0.176
**EQ5D-3L domains**								
Mobility							0.73 (0.27-2.02)	0.546
Self-care							1.33 (0.41-4.32)	0.634
Usual activities							1.93 (0.76-4.89)	0.168
Pain/discomfort							1.41 (0.89-2.21)	0.142

Note:
Model 1: Adjusted for age, gender, occupation status, highest education attained, smoking status, duration of diabetes, BMI, systolic and diastolic blood pressure, HbA1c, lipid profile (LDL cholesterol, HDL cholesterol, triglycerides).
Model 2: Model 1 + adjusted for Ln(ACR+1) , eGFR, use of lipid lowering drugs, ACEI or ARB, other anti-hypertensive drugs, anti-diabetic drugs, insulin

Model 3: Model 2 + adjusted for adherence to balanced diet, physical activity, level of medication adherence and self-monitoring of blood glucose in last 3 months and regular follow up in last 1 year
Model 4: Model 3 + adjusted for ED-5D-3L Q1-Q4 (excluding Q5 on anxiety/ depression)
[a] *Including IHD*
[b] *Models 1-4 for CKD outcome excluded adjustment for Ln(urine ACR+1) and eGFR*
Bold values represent $p<0.05$

Supplementary table 6. Stepwise logistic regression for CKD in Chinese patients with T2D (*n* = 533)

	Model 1		Model 2		Model 3		Model 4	
	HR (95% CI)	P-value	HR (95% CI)	P-value	HR (95% CI)	P-value	HR (95% CI)	P-value
PHQ-9 score ≥ 7	1.24 (0.96-1.60)	0.101	1.22 (0.95-1.58)	0.126	1.14 (0.87-1.51)	0.344	1.04 (0.77-1.40)	0.820
**Demographics**								
Age (years)	1.06 (1.04-1.07)	**<0.001**	1.05 (1.04-1.07)	**<0.001**	1.06 (1.04-1.07)	**<0.001**	1.05 (1.04-1.07)	**<0.001**
Male gender (ref: female)	1.75 (1.38-2.20)	**<0.001**	1.79 (1.41-2.25)	**<0.001**	1.80 (1.41-2.30)	**<0.001**	1.84 (1.44-2.37)	**<0.001**
Employed (ref: unemployed)	0.72 (0.58-0.90)	**0.003**	0.74 (0.59-0.92)	**0.006**	0.72 (0.57-0.91)	**0.005**	0.74 (0.58-0.94)	**0.012**
Highest education attained (ref: primary, illiterate and others)								
Middle school	0.88 (0.71-1.08)	0.225	0.88 (0.71-1.08)	0.215	0.92 (0.74-1.16)	0.489	0.93 (0.75-1.17)	0.538
Higher school	0.73 (0.50-1.06)	0.100	0.76 (0.52-1.10)	0.143	0.87 (0.59-1.28)	0.476	0.89 (0.60-1.30)	0.534
College or above	0.74 (0.55-1.00)	**0.048**	0.77 (0.57-1.03)	0.078	0.83 (0.61-1.14)	0.254	0.85 (0.62-1.16)	0.305
Smoking status (ref: non-smoker)								
Ex-smoker	0.90 (0.71-1.15)	0.406	0.91 (0.71-1.16)	0.421	0.90 (0.69-1.16)	0.409	0.89 (0.68-1.15)	0.372
Current smoker	0.84 (0.61-1.16)	0.287	0.84 (0.61-1.16)	0.280	0.92 (0.66-1.29)	0.641	0.92 (0.65-1.28)	0.607
Duration of diabetes (years)	1.02 (1.01-1.04)	**<0.001**	1.01 (1.00-1.02)	0.141	1.01 (1.00-1.03)	**0.047**	1.01 (1.00-1.03)	0.051
**Clinical parameters**								
Body mass index (kg/m^2)	1.02 (1.00-1.04)	0.084	1.00 (0.98-1.02)	0.807	1.00 (0.98-1.02)	0.958	1.00 (0.97-1.02)	0.804
Systolic blood pressure (mmHg)	1.02 (1.01-1.03)	**<0.001**	1.02 (1.01-1.02)	**<0.001**	1.02 (1.01-1.02)	**<0.001**	1.02 (1.01-1.02)	**<0.001**
Diastolic blood pressure (mmHg)	1.00 (0.99-1.01)	0.757	1.00 (0.99-1.01)	0.965	1.00 (0.99-1.01)	0.830	1.00 (0.99-1.01)	0.904
HbA1c (%)	1.11 (1.05-1.18)	**<0.001**	1.08 (1.02-1.16)	**0.016**	1.09 (1.02-1.17)	**0.017**	1.09 (1.01-1.17)	**0.020**
LDL-cholesterol (mmol/L)	0.92 (0.82-1.05)	0.220	1.01 (0.88-1.15)	0.905	0.98 (0.85-1.13)	0.771	0.97 (0.84-1.12)	0.647
HDL-cholesterol (mmol/L)	0.71 (0.53-0.94)	**0.017**	0.79 (0.59-1.05)	0.103	0.76 (0.56-1.03)	0.081	0.77 (0.57-1.05)	0.094
Triglyceride (mmol/L)	1.16 (1.06-1.25)	**<0.001**	1.21 (1.10-1.32)	**<0.001**	1.21 (1.09-1.34)	**<0.001**	1.20 (1.08-1.33)	**<0.001**
**Drug use at enrolment (ref: not on respective drug)**								
Lipid lowering drugs			1.04 (0.86-1.27)	0.688	1.00 (0.81-1.24)	0.971	1.00 (0.81-1.23)	1.000
Antihypertensive drugs, including ACEIs or ARBs			2.63 (1.97-3.51)	**<0.001**	2.32 (1.71-3.14)	**<0.001**	2.32 (1.71-3.14)	**<0.001**
Oral anti-diabetic drugs			1.10 (0.78-1.54)	0.588	0.96 (0.66-1.42)	0.854	0.97 (0.66-1.43)	0.869

Insulin			1.41 (1.13-1.76)	**0.003**	1.33 (1.05-1.68)	**0.020**	1.30 (1.02-1.65)	**0.031**
**Diabetes self-care**								
Self-reported level of medication adherence (0-100)					1.00 (0.99-1.01)	0.464	1.00 (0.99-1.01)	0.588
Regular physical activity in last 3 months (ref: no regular physical activity)								
1-3 times per week					0.78 (0.58-1.04)	0.087	0.79 (0.59-1.06)	0.112
3-4 times per week					0.60 (0.41-0.88)	**0.009**	0.61 (0.41-0.89)	**0.011**
5 times per week					0.87 (0.54-1.42)	0.583	0.88 (0.54-1.42)	0.591
> 5 times per week					0.89 (0.70-1.11)	0.294	0.90 (0.71-1.13)	0.345
Adherence to a balanced diet in last 3 months (ref: never)								
No					0.61 (0.31-1.20)	0.154	0.60 (0.30-1.19)	0.144
Occasional					0.73 (0.40-1.32)	0.300	0.71 (0.39-1.28)	0.249
Yes					0.76 (0.42-1.37)	0.366	0.74 (0.41-1.33)	0.308
Self-monitoring of glucose (ref: none)					1.13 (0.86-1.48)	0.389	1.13 (0.86-1.48)	0.397
Regular follow up (ref: none)					2.67 (0.85-8.39)	0.094	2.59 (0.82-8.16)	0.103
**EQ5D-3L domains**								
Mobility							1.11 (0.75-1.65)	0.597
Self-care							0.55 (0.28-1.05)	0.071
Usual activities							1.37 (0.91-2.08)	0.133
Pain/discomfort							1.15 (0.95-1.39)	0.144

Note:
Model 1: Adjusted for age, gender, occupation status, highest education attained, smoking status, duration of diabetes, BMI, systolic and diastolic blood pressure, HbA1c, lipid profile (LDL cholesterol, HDL cholesterol, triglycerides).
Model 2: Model 1 + adjusted for Ln(ACR+1) , eGFR, use of lipid lowering drugs, ACEI or ARB, other anti-hypertensive drugs, anti-diabetic drugs, insulin
Model 3: Model 2 + adjusted for adherence to balanced diet, physical activity, level of medication adherence and self-monitoring of blood glucose in last 3 months and regular follow up in last 1 year
Model 4: Model 3 + adjusted for ED-5D-3L Q1-Q4 (excluding Q5 on anxiety/ depression)
[a] *(Including IHD)*
[b] *Models 1-4 for CKD outcome excluded adjustment for Ln(urine ACR+1) and eGFR*

Bold values represent $p<0.05$

Supplementary table 7. Logistic regression for all-cause mortality in Chinese patients with type 2 diabetes, using PHQ-2≥3 as cut-off (*n* = 141)

	PHQ-2 model 4		PHQ-9 model 4	
	**HR (95% CI)**	**P-value**	**HR (95% CI)**	**P-value**
PHQ-2 score ≥ 3/ PHQ-9 score ≥ 7	1.51 (0.73-3.13)	0.269	1.54 (0.91-2.60)	0.108
**Demographics**				
Age (years)	1.08 (1.05-1.11)	**<0.001**	1.08 (1.05-1.11)	**<0.001**
Male gender (ref: female)	1.14 (0.68-1.93)	0.612	1.13 (0.67-1.90)	0.656
Employed (ref: unemployed)	0.62 (0.39-0.99)	**0.046**	0.63 (0.40-1.01)	0.057
Highest education attained (ref: primary, illiterate and others)				
Middle school	1.10 (0.71-1.70)	0.683	1.12 (0.72-1.73)	0.626
Higher school	1.12 (0.55-2.30)	0.751	1.15 (0.56-2.34)	0.709
College or above	1.15 (0.63-2.12)	0.652	1.18 (0.64-2.19)	0.588
Smoking status (ref: non-smoker)				
Ex-smoker	2.58 (1.58-4.22)	**<0.001**	2.61 (1.60-4.28)	**<0.001**
Current smoker	2.06 (1.11-3.84)	**0.023**	2.12 (1.14-3.95)	**0.018**
Duration of diabetes (years)	1.01 (0.99-1.04)	0.365	1.01 (0.99-1.04)	0.348
**Clinical parameters**				
Body mass index (kg/m^2)	0.98 (0.93-1.03)	0.336	0.98 (0.93-1.03)	0.374
Systolic blood pressure (mmHg)	1.01 (1.00-1.02)	0.199	1.01 (1.00-1.02)	0.181
Diastolic blood pressure (mmHg)	1.00 (0.98-1.02)	0.972	1.00 (0.98-1.02)	0.951
HbA1c (%)	1.30- (1.17-1.45)	**<0.001**	1.29 (1.15-1.43)	**<0.001**
LDL-cholesterol (mmol/L)	0.81 (0.61-1.07)	0.141	0.81 (0.61-1.08)	0.147
HDL-cholesterol (mmol/L)	0.90 (0.53-1.53)	0.708	0.91 (0.54-1.54)	0.725
Triglyceride (mmol/L)	0.80 (0.59-1.09)	0.162	0.81 (0.60-1.10)	0.177
Ln(urine ACR + 1)	1.22 (1.05-1.41)	**0.008**	1.21 (1.05-1.40)	**0.009**
eGFR (mL/min/1.73 m^2)	1.02 (1.00-1.04)	0.069	1.02 (1.00-1.04)	0.068
**Drug use at enrolment (ref: not on respective drug)**				
Lipid lowering drugs	0.77 (0.51-1.14)	0.194	0.77 (0.52-1.15)	0.199

Antihypertensive drugs, including ACEIs or ARBs	0.99 (0.61-1.61)	0.960	0.97 (0.60-1.59)	0.910
Oral anti-diabetic drugs	0.49 (0.27-0.88)	**0.018**	0.49 (0.27-0.89)	**0.018**
Insulin	0.85 (0.53-1.37)	0.508	0.85 (0.53-1.37)	0.511
**Diabetes self-care**				
Self-reported level of medication adherence (0-100)	1.00 (0.98-1.01)	0.658	1.00 (0.98-1.01)	0.689
Regular physical activity in last 3 months (ref: no regular physical activity)				
1-3 times per week	0.88 (0.48-1.60)	0.677	0.90 (0.49-1.64)	0.729
3-4 times per week	0.73 (0.35-1.52)	0.400	0.76 (0.36-1.59)	0.460
5 times per week	1.22 (0.51-2.96)	0.656	1.28 (0.53-3.11)	0.585
> 5 times per week	0.81 (0.52-1.29)	0.377	0.85 (0.54-1.35)	0.496
Adherence to a balanced diet in last 3 months (ref: never)				
No	1.38 (0.38-4.97)	0.623	1.39 (0.39-5.01)	0.616
Occasional	0.81 (0.25-2.68)	0.730	0.79 (0.24-2.59)	0.691
Yes	1.03 (0.31-3.37)	0.964	1.00 (0.31-3.27)	1.000
Self-monitoring of glucose (ref: none)	0.80 (0.49-1.30)	0.371	0.79 (0.49-1.29)	0.349
Regular follow up (ref: none)	1.64 (0.39-6.88)	0.498	1.63 (0.39-6.87)	0.504
**EQ5D-3L domains**				
Mobility	0.82 (0.38-1.76)	0.609	0.81 (0.38-1.73)	0.591
Self-care	1.43 (0.58-3.53)	0.434	1.46 (0.59-3.58)	0.412
Usual activities	1.54 (0.77-3.10)	0.223	1.50 (0.75-3.00)	0.257
Pain/discomfort	1.24 (0.87-1.78)	0.241	1.21 (0.84-1.73)	0.310

Note:
Model 1: Adjusted for age, gender, occupation status, highest education attained, smoking status, duration of diabetes, BMI, systolic and diastolic blood pressure, HbA1c, lipid profile (LDL cholesterol, HDL cholesterol, triglycerides).
Model 2: Model 1 + adjusted for Ln(ACR+1) , eGFR, use of lipid lowering drugs, ACEI or ARB, other anti-hypertensive drugs, anti-diabetic drugs, insulin
Model 3: Model 2 + adjusted for adherence to balanced diet, physical activity, level of medication adherence and self-monitoring of blood glucose in last 3 months and regular follow up in last 1 year
Model 4: Model 3 + adjusted for ED-5D-3L Q1-Q4 (excluding Q5 on anxiety/ depression)
[a] *Including IHD*

[b] *Models 1-4 for CKD outcome excluded adjustment for Ln(urine ACR+1) and eGFR*
Bold values represent p<0.05

Supplementary table 8. Logistic regression for all CVD outcomes (including IHD) in Chinese patients with type 2 diabetes, using PHQ-2 ≥3 as cut-off (*n* = 164)

	PHQ-2 model 4		PHQ-9 model 4	
	HR (95% Cl)	P-value	HR (95% Cl)	P-value
PHQ-2 score ≥ 3/ PHQ-9 score ≥ 7	2.67 (1.47-4.85)	**0.001**	1.99 (1.25-3.18)	**0.004**
**Demographics**				
Age (years)	1.04 (1.01-1.07)	**0.009**	1.04 (1.01-1.07)	**0.006**
Male gender (ref: female)	2.06 (1.26-3.37)	**0.004**	2.04 (1.25-3.33)	**0.005**
Employed (ref: unemployed)	1.18 (0.78-1.78)	0.444	1.22 (0.80-1.84)	0.361
Highest education attained (ref: primary, illiterate and others)				
Middle school	1.36 (0.88-2.12)	0.168	1.40 (0.90-2.18)	0.141
Higher school	1.28 (0.64-2.55)	0.478	1.32 (0.66-2.62)	0.436
College or above	1.25 (0.69-2.26)	0.458	1.29 (0.71-2.33)	0.404
Smoking status (ref: non-smoker)				
Ex-smoker	1.30 (0.81-2.11)	0.279	1.30 (0.80-2.10)	0.290
Current smoker	2.27 (1.42-3.65)	**<0.001**	2.34 (1.45-3.77)	**<0.001**
Duration of diabetes (years)	1.01 (0.99-1.04)	0.284	1.01 (0.99-1.04)	0.310
**Clinical parameters**				
Body mass index (kg/m^2)	0.98 (0.94-1.03)	0.386	0.98 (0.94-1.03)	0.438
Systolic blood pressure (mmHg)	1.01 (1.00-1.02)	0.160	1.01 (1.00-1.02)	0.162
Diastolic blood pressure (mmHg)	1.01 (0.99-1.03)	0.483	1.01 (0.99-1.03)	0.452
HbA1c (%)	1.04 (0.92-1.18)	0.491	1.04 (0.92-1.17)	0.575
LDL-cholesterol (mmol/L)	1.37 (1.09-1.73)	**0.007**	1.37 (1.09-1.72)	**0.008**
HDL-cholesterol (mmol/L)	1.16 (0.69-1.93)	0.578	1.19 (0.71-2.00)	0.509
Triglyceride (mmol/L)	1.19 (1.00-1.41)	**0.048**	1.20 (1.01-1.43)	**0.040**
Ln(urine ACR + 1)	1.14 (0.99-1.30)	0.063	1.13 (0.98-1.29)	0.085
eGFR (mL/min/1.73 m^2)	0.99 (0.97-1.00)	0.153	0.99 (0.98-1.01)	0.179
**Drug use at enrolment (ref: not on respective drug)**				

Lipid lowering drugs	1.00 (0.69-1.46)	0.989	1.02 (0.70-1.48)	0.929
Antihypertensive drugs, including ACEIs or ARBs	1.06 (0.68-1.67)	0.789	1.05 (0.67-1.66)	0.820
Oral anti-diabetic drugs	1.04 (0.51-2.12)	0.905	1.01 (0.50-2.04)	0.988
Insulin	1.47 (0.95-2.27)	0.087	1.49 (0.97-2.31)	0.072
**Diabetes self-care**				
Self-reported level of medication adherence (0-100)	0.99 (0.98-1.00)	0.206	0.99 (0.98-1.01)	0.230
Regular physical activity in last 3 months (ref: no regular physical activity)				
1-3 times per week	0.96 (0.60-1.54)	0.856	0.98 (0.61-1.57)	0.925
3-4 times per week	0.49 (0.23-1.04)	0.063	0.51 (0.24-1.08)	0.080
5 times per week	0.53 (0.16-1.71)	0.288	0.54 (0.17-1.75)	0.306
> 5 times per week	0.68 (0.44-1.06)	0.088	0.72 (0.46-1.12)	0.144
Adherence to a balanced diet in last 3 months (ref: never)				
No	0.83 (0.27-2.63)	0.756	0.82 (0.26-2.57)	0.730
Occasional	0.72 (0.26-2.04)	0.537	0.70 (0.25-1.97)	0.495
Yes	0.83 (0.29-2.33)	0.717	0.80 (0.28-2.25)	0.668
Self-monitoring of glucose (ref: none)	1.08 (0.66-1.78)	0.754	1.10 (0.67-1.81)	0.707
Regular follow up (ref: none)	0.64 (0.26-1.62)	0.349	0.63 (0.25-1.60)	0.332
**EQ5D-3L domains**				
Mobility	0.56 (0.24-1.32)	0.186	0.54 (0.23-1.25)	0.149
Self-care	0.93 (0.31-2.74)	0.892	1.00 (0.34-2.92)	0.993
Usual activities	1.91 (0.92-3.97)	0.083	1.91 (0.93-3.92)	0.080
Pain/discomfort	1.05 (0.74-1.49)	0.776	1.06 (0.75-1.50)	0.740

Note:
Model 1: Adjusted for age, gender, occupation status, highest education attained, smoking status, duration of diabetes, BMI, systolic and diastolic blood pressure, HbA1c, lipid profile (LDL cholesterol, HDL cholesterol, triglycerides).
Model 2: Model 1 + adjusted for Ln(ACR+1) , eGFR, use of lipid lowering drugs, ACEI or ARB, other anti-hypertensive drugs, anti-diabetic drugs, insulin
Model 3: Model 2 + adjusted for adherence to balanced diet, physical activity, level of medication adherence and self-monitoring of blood glucose in last 3 months and regular follow up in last 1 year

Model 4: Model 3 + adjusted for ED-5D-3L Q1-Q4 (excluding Q5 on anxiety/ depression)
[a] *Including IHD*
[b] *Models 1-4 for CKD outcome excluded adjustment for Ln(urine ACR+1) and eGFR*
Bold values represent p<0.05

Supplementary table 9. Logistic regression for IHD in Chinese patients with type 2 diabetes, using PHQ-2≥3 as cut-off (*n* = 95)

	PHQ-2 model 4	P-value	PHQ-9 model 4	P-value
	HR (95% CI)		HR (95% CI)	
PHQ-2 score ≥ 3/ PHQ-9 score ≥ 7	1.8 (0.79-4.47)	0.154	2.29 (1.25-4.21)	**0.008**
**Demographics**				
Age (years)	1.03 (0.99-1.07)	0.132	1.03 (0.99-1.07)	0.095
Male gender (ref: female)	3.56 (1.73-7.32)	**<0.001**	3.43 (1.67-7.05)	**<0.001**
Employed (ref: unemployed)	1.07 (0.61-1.85)	0.822	1.14 (0.65-1.99)	0.645
Highest education attained (ref: primary, illiterate and others)				
Middle school	1.61 (0.86-3.04)	0.140	1.66 (0.88-3.14)	0.118
Higher school	1.91 (0.80-4.53)	0.143	2.02 (0.85-4.81)	0.113
College or above	1.58 (0.71-3.52)	0.267	1.61 (0.72-3.61)	0.250
Smoking status (ref: non-smoker)				
Ex-smoker	1.47 (0.79-2.72)	0.221	1.49 (0.80-2.77)	0.205
Current smoker	2.60 (1.39-4.86)	**0.003**	2.67 (1.43-4.99)	**0.002**
Duration of diabetes (years)	1.04 (1.00-1.08)	**0.026**	1.04 (1.00-1.07)	**0.035**
**Clinical parameters**				
Body mass index (kg/m^2)	1.00 (0.94-1.06)	0.913	1.00 (0.94-1.06)	0.959
Systolic blood pressure (mmHg)	1.01 (1.00-1.03)	0.140	1.01 (1.00-1.03)	0.134
Diastolic blood pressure (mmHg)	0.98 (0.96-1.01)	0.260	0.98 (0.95-1.01)	0.250
HbA1c (%)	0.98 (0.83-1.17)	0.861	0.98 (0.82-1.16)	0.810
LDL-cholesterol (mmol/L)	1.47 (1.07-2.01)	**0.017**	1.46 (1.07-1.99)	**0.018**
HDL-cholesterol (mmol/L)	1.01 (0.49-2.05)	0.988	1.03 (0.50-2.11)	0.936
Triglyceride (mmol/L)	1.20 (0.94-1.54)	0.142	1.22 (0.96-1.55)	0.113
Ln(urine ACR + 1)	1.15 (0.96-1.38)	0.128	1.15 (0.95-1.37)	0.147
eGFR ($mL/min/1.73\ m^2$)	1.00 (0.98-1.02)	0.695	1.00 (0.98-1.02)	0.691
**Drug use at enrolment (ref: not on respective drug)**				
Lipid lowering drugs	1.13 (0.68-1.88)	0.626	1.13 (0.68-1.87)	0.640
Antihypertensive drugs, including ACEIs or ARBs	0.96 (0.52-1.75)	0.884	0.94 (0.51-1.72)	0.840

Oral anti-diabetic drugs	1.82 (0.55-5.03)	0.329	1.76 (0.53-5.83)	0.357
Insulin	1.01 (0.56-1.83)	0.972	1.04 (0.58-1.88)	0.891
**Diabetes self-care**				
Self-reported level of medication adherence (0-100)	1.00 (0.98-1.02)	0.913	1.00 (0.98-1.02)	0.934
Regular physical activity in last 3 months (ref: no regular physical activity)				
1-3 times per week	1.56 (0.85-2.86)	0.155	1.64 (0.89-3.03)	0.116
3-4 times per week	0.41 (0.12-1.40)	0.155	0.43 (0.13-1.47)	0.178
5 times per week	0.40 (0.05-2.99)	0.372	0.41 (0.06-3.09)	0.390
> 5 times per week	0.92 (0.50-1.67)	0.771	0.99 (0.54-1.81)	0.967
Adherence to a balanced diet in last 3 months (ref: never)				
No	0.90 (0.10-7.97)	0.926	0.93 (0.11-8.23)	0.950
Occasional	1.55 (0.21-11.6)	0.688	1.50 (0.20-11.2)	0.690
Yes	1.49 (0.20-11.1)	0.700	1.42 (0.19-10.6)	0.731
Self-monitoring of glucose (ref: none)	1.01 (0.52-1.95)	0.984	1.00 (0.51-1.94)	0.996
Regular follow up (ref: none)	0.46 (0.14-1.53)	0.205	0.44 (0.13-1.45)	0.176
**EQ5D-3L domains**				
Mobility	0.76 (0.27-2.15)	0.606	0.73 (0.27-2.02)	0.546
Self-care	1.35 (0.42-4.37)	0.615	1.33 (0.41-4.32)	0.634
Usual activities	2.15 (0.84-5.51)	0.110	1.93 (0.76-4.89)	0.168
Pain/discomfort	1.49 (0.95-2.33)	0.081	1.41 (0.89-2.21)	0.142

Note:
Model 1: Adjusted for age, gender, occupation status, highest education attained, smoking status, duration of diabetes, BMI, systolic and diastolic blood pressure, HbA1c, lipid profile (LDL cholesterol, HDL cholesterol, triglycerides).
Model 2: Model 1 + adjusted for Ln(ACR+1) , eGFR, use of lipid lowering drugs, ACEI or ARB, other anti-hypertensive drugs, anti-diabetic drugs, insulin
Model 3: Model 2 + adjusted for adherence to balanced diet, physical activity, level of medication adherence and self-monitoring of blood glucose in last 3 months and regular follow up in last 1 year
Model 4: Model 3 + adjusted for ED-5D-3L Q1-Q4 (excluding Q5 on anxiety/ depression)
[a] *Including IHD*
[b] *Models 1-4 for CKD outcome excluded adjustment for Ln(urine ACR+1) and eGFR*
Bold values represent p<0.05

Supplementary table 10. Logistic regression for CKD in Chinese patients with type 2 diabetes, using PHQ-2≥3 as cut-off (*n* = 533)

	PHQ-2 model 4	P-value	PHQ-9 model 4	P-value
	HR (95% CI)		HR (95% CI)	
PHQ-2 score ≥ 3/ PHQ-9 score ≥ 7	1.35 (0.90-2.04)	0.150	1.04 (0.77-1.40)	0.820
**Demographics**				
Age (years)	1.06 (1.04-1.07)	**<0.001**	1.05 (1.04-1.07)	**<0.001**
Male gender (ref: female)	1.86 (1.45-2.38)	**<0.001**	1.84 (1.44-2.37)	**<0.001**
Employed (ref: unemployed)	0.74 (0.58-0.94)	**0.013**	0.74 (0.58-0.94)	**0.012**
Highest education attained (ref: primary, illiterate and others)				
Middle school	0.93 (0.74-1.16)	0.512	0.93 (0.75-1.17)	0.538
Higher school	0.89 (0.61-1.30)	0.539	0.89 (0.60-1.30)	0.534
College or above	0.85 (0.62-1.16)	0.314	0.85 (0.62-1.16)	0.305
Smoking status (ref: non-smoker)				
Ex-smoker	0.89 (0.69-1.15)	0.379	0.89 (0.68-1.15)	0.372
Current smoker	0.91 (0.65-1.28)	0.586	0.92 (0.65-1.28)	0.607
Duration of diabetes (years)	1.01 (1.00-1.03)	**0.043**	1.01 (1.00-1.03)	0.051
**Clinical parameters**				
Body mass index (kg/m^2)	1.00 (0.97-1.02)	0.814	1.00 (0.97-1.02)	0.804
Systolic blood pressure (mmHg)	1.02 (1.01-1.02)	**<0.001**	1.02 (1.01-1.02)	**<0.001**
Diastolic blood pressure (mmHg)	1.00 (0.99-1.01)	0.880	1.00 (0.99-1.01)	0.904
HbA1c (%)	1.09 (1.01-1.17)	**0.022**	1.09 (1.01-1.17)	**0.020**
LDL-cholesterol (mmol/L)	0.97 (0.84-1.12)	0.675	0.97 (0.84-1.12)	0.647
HDL-cholesterol (mmol/L)	0.76 (0.56-1.04)	0.082	0.77 (0.57-1.05)	0.094
Triglyceride (mmol/L)	1.19 (1.08-1.32)	**<0.001**	1.20 (1.08-1.33)	**<0.001**
**Drug use at enrolment (ref: not on respective drug)**				
Lipid lowering drugs	1.00 (0.81-1.23)	0.998	1.00 (0.81-1.23)	1.000
Antihypertensive drugs, including ACEIs or ARBs	2.32 (1.71-3.15)	**<0.001**	2.32 (1.71-3.14)	**<0.001**
Oral anti-diabetic drugs	0.97 (0.66-1.43)	0.887	0.97 (0.66-1.43)	0.869
Insulin	1.29 (1.02-1.64)	**0.035**	1.30 (1.02-1.65)	**0.031**

**Diabetes self-care**				
Level of medication adherence (0-100)	1.00 (0.99-1.01)	0.606	1.00 (0.99-1.01)	0.588
Regular physical activity in last 3 months (ref: no regular physical activity)				
1-3 times per week	0.79 (0.59-1.06)	0.113	0.79 (0.59-1.06)	0.112
3-4 times per week	0.60 (0.41-0.89)	**0.011**	0.61 (0.41-0.89)	**0.011**
5 times per week	0.89 (0.55-1.44)	0.620	0.88 (0.54-1.42)	0.591
> 5 times per week	0.90 (0.71-1.13)	0364	0.90 (0.71-1.13)	0.345
Adherence to a balanced diet in last 3 months (ref: never)				
No	0.60 (0.30-1.18)	0.136	0.60 (0.30-1.19)	0.144
Occasional	0.71 (0.39-1.28)	0.257	0.71 (0.39-1.28)	0.249
Yes	0.74 (0.41-1.55)	0.312	0.74 (0.41-1.33)	0.308
Self-monitoring of glucose (ref: none)	1.12 (0.85-1.48)	0.407	1.13 (0.86-1.48)	0.397
Regular follow up (ref: none)	2.57 (0.82-8.08)	0.107	2.59 (0.82-8.16)	0.103
**EQ5D-3L domains**				
Mobility	1.11 (0.75-1.64)	0.617	1.11 (0.75-1.65)	0.597
Self-care	0.53 (0.27-1.02)	0.059	0.55 (0.28-1.05)	0.071
Usual activities	1.33 (0.88-2.01)	0.176	1.37 (0.91-2.08)	0.133
Pain/discomfort	1.14 (0.94-1.37)	0.181	1.15 (0.95-1.39)	0.144

Note:
Model 1: Adjusted for age, gender, occupation status, highest education attained, smoking status, duration of diabetes, BMI, systolic and diastolic blood pressure, HbA1c, lipid profile (LDL cholesterol, HDL cholesterol, triglycerides).
Model 2: Model 1 + adjusted for Ln(ACR+1) , eGFR, use of lipid lowering drugs, ACEI or ARB, other anti-hypertensive drugs, anti-diabetic drugs, insulin
Model 3: Model 2 + adjusted for adherence to balanced diet, physical activity, level of medication adherence and self-monitoring of blood glucose in last 3 months and regular follow up in last 1 year
Model 4: Model 3 + adjusted for ED-5D-3L Q1-Q4 (excluding Q5 on anxiety/ depression)
[a] *Including IHD*
[b] *Models 1-4 for CKD outcome excluded adjustment for Ln(urine ACR+1) and eGFR*
Bold values represent p<0.05
